# Three-dimensional non-destructive visualization of teeth enamel microcracks using X-ray micro-computed tomography

**DOI:** 10.1038/s41598-021-94303-4

**Published:** 2021-07-20

**Authors:** Irma Dumbryte, Arturas Vailionis, Edvinas Skliutas, Saulius Juodkazis, Mangirdas Malinauskas

**Affiliations:** 1 Vilnius University, Vilnius, Lithuania; 2 Stanford Nano Shared Facilities, Stanford University, Stanford, USA; 3 Department of Physics, Kaunas University of Technology, Kaunas, Lithuania; 4 Laser Research Center, Faculty of Physics, Vilnius University, Vilnius, Lithuania; 5 Optical Sciences Centre and ARC Training Centre in Surface Engineering for Advanced Materials (SEAM), School of Science, Swinburne University of Technology, Hawthorn, Australia; 6 Tokyo Tech World Research Hub Initiative (WRHI), School of Materials and Chemical Technology, Tokyo Institute of Technology, Tokyo, Japan

**Keywords:** X-rays, X-ray tomography, Enamel

## Abstract

Although the topic of tooth fractures has been extensively analyzed in the dental literature, there is still insufficient information about the potential effect of enamel microcracks (EMCs) on the underlying tooth structures. For a precise examination of the extent of the damage to the tooth structure in the area of EMCs, it is necessary to carry out their volumetric [(three-dimensional (3D)] evaluation. The aim of this study was to validate an X-ray micro-computed tomography ($\mu$CT) as a technique suitable for 3D non-destructive visualization and qualitative analysis of teeth EMCs of different severity. Extracted human maxillary premolars were examined using a $\mu$CT instrument ZEISS Xradia 520 Versa. In order to separate crack, dentin, and enamel volumes a Deep Learning (DL) algorithm, part of the Dragonfly’s segmentation toolkit, was utilized. For segmentation needs we implemented Dragonfly’s pre-built UNet neural network. The scanning technique which was used made it possible to recognize and detect not only EMCs that are visible on the outer surface but also those that are buried deep inside the tooth. The 3D visualization, combined with DL assisted segmentation, enabled the evaluation of the dynamics of an EMC and precise examination of its position with respect to the dentin-enamel junction.

## Introduction

**Figure 1:**
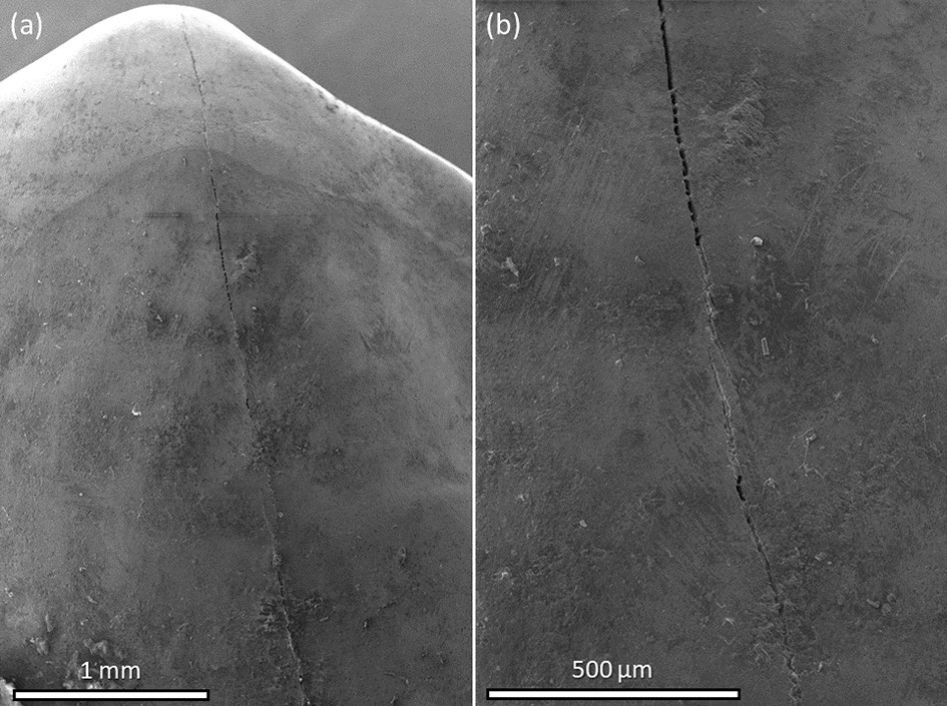
An example of SEM micrographs of EMCs on the buccal enamel surface: (**a**) the occlusal and (**b**) the middle part of the tooth crown.

A careful evaluation of teeth with an intense light source during a routine dental examination can often reveal microcracks in the enamel. They can be described as enamel microcracks (EMCs) that usually do not cross the dentin-enamel junction (DEJ) and have no loss or visible separation of tooth structure^1–5^ (typical images of EMCs using a scanning electron microscope [(SEM, Fischer Scientific The Prisma E, operated at 5 kV, 0.18 nA (beam current)] demonstrated in Fig. 1). These EMCs have also been categorized as incomplete tooth fractures (ITFs) and the following definition has been proposed: “*a fracture plane of unknown depth and direction passing through tooth structure that, if not already involving, may progress to communicate with the pulp and/or periodontal ligament*”^5^. The formation of EMCs has generally been attributed to the abnormalities in the maturation process, occlusal forces (e.g. those generated during dental attrition and abrasion), traumatic injuries (e.g. provoked by the tooth extraction procedure), temperature variations, and restorative processes^3,6–9^. As previously published studies have demonstrated, EMCs can be noticed after brackets’ removal at the end of the orthodontic treatment^6,10–14^.

Although the topic of tooth fractures has been extensively analyzed in the dental literature, there is still insufficient information about the potential effect of EMCs on the underlying tooth structures. Over the last two decades an increasing number of scientific reports have been published, examining the qualitative and quantitative characteristics of EMCs (e.g. their number, direction, location, length, and width) and their changes following orthodontic debonding^10,12,15–20^. However, the evaluation of the above parameters provides only a lateral [(two-dimensional (2D)] characterization of the enamel surface. For a more precise examination of the damage caused to the tooth structure in the area of EMCs (i.e. whether an EMC crosses the DEJ and reaches the dentin or the pulp), a volumetric [three-dimensional (3D)] visualization of EMCs is necessary. Several attempts to measure the depth parameter should be mentioned^21–23^. However, the limitations of the techniques used in those studies—notably, the limited penetration depth and scanning range of the device utilized for examination of different parameters of EMCs^21,23^, sensitivity of the technique to non-flat surfaces^22,23^ or the depth measurements carried out on a simulated human tooth^22^—have prompted us to look for a new 3D method that would enable a non-destructive evaluation of EMCs with micrometer resolution.

Advances in optical microscopy are driven by widespread sophisticated additive manufacturing techniques^24^ enabling miniaturization of equipment footprint suitable for minimal invasive procedures^25^. In parallel, multi-modal imaging can be obtained at the same time by acquiring absorbance (dichroism) and birefringence^26^ or simultaneus spatio-spectral-temporal mapping^27^. However, to date it has been possible to achieve those results only in the laboratory environment and not in clinical practice.

X-ray micro-computed tomography ($\mu$CT) is a 3D imaging technique that utilizes X-rays to see inside an object. It is an accurate, non-destructive method that provides volumetric information about the microstructure ( today’s most advanced laboratory-based $\mu$CTs can achieve resolutions up to 0.7–${1}{\, \upmu \hbox {m}}$ (4–${10}{\, \upmu \hbox {m}}$ resolution usually selected for biological samples) using geometrical magnification) and generally does not require extensive sample preparation^28^. This provides an advantage over other EMCs’ examination techniques which have been used to date, such as stereomicroscopy^12,13,15,16^, SEM^10,29^, or 3D scanning methods (optical coherence tomography (OCT) and ultrasound)^21,22,30–32^. Employing $\mu$CT in dental studies is of increasing interest, including its application in enamel thickness and tooth measurements, caries research, characterization of enamel white spot lesions and cortical bone microdamage, root canal morphology and preparation analysis, detection of various types of teeth fractures, and dental tissue engineering^33–41^. Despite the great potential of this technique, no studies utilizing $\mu$CT to evaluate the inner tooth structure in the area of EMCs of human teeth have been reported to date.

Thus, the aim of this in vitro study was to validate an X-ray $\mu$CT as a technique suitable for 3D non-destructive visualization and qualitative analysis of EMCs. An additional objective was to demonstrate that this method can be used for the recognition and detection of EMCs of different severity (i.e. those which are visible on the outer surface and those that are buried deep inside the tooth).

## Methods

**Figure 2:**
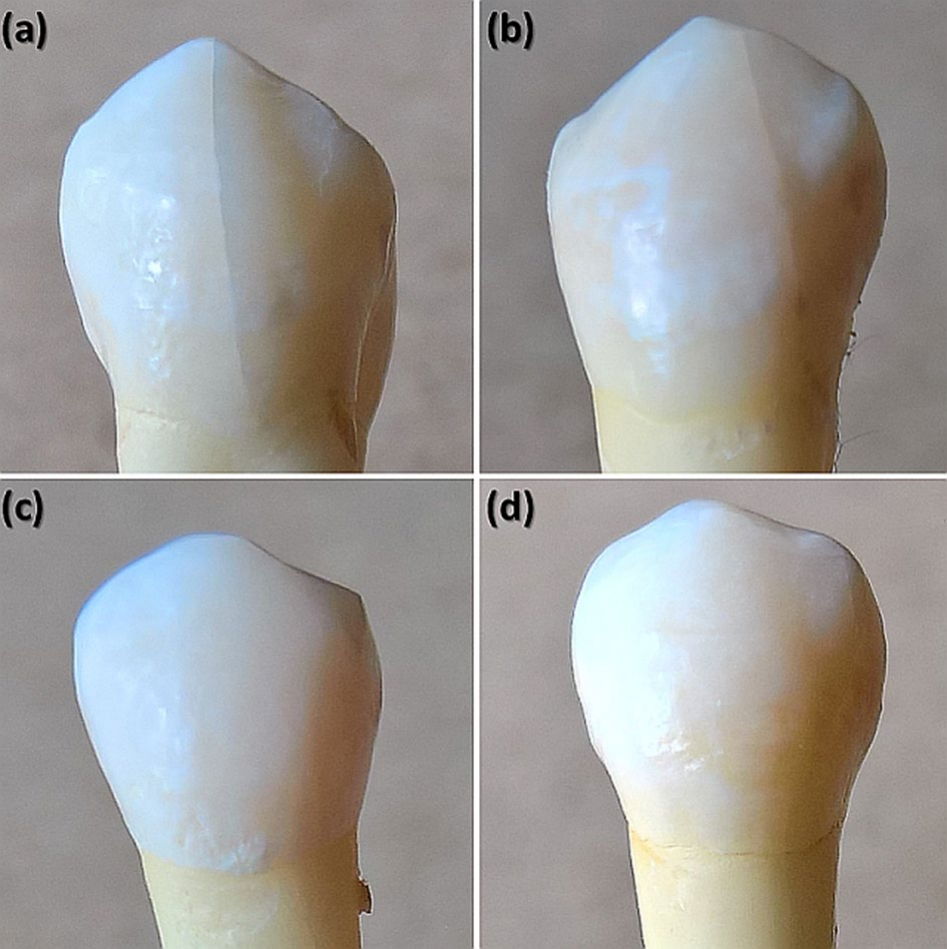
An example of photographs of teeth with (**a**,**b**) visible and (**c**,**d**) invisible EMCs on the outer buccal tooth surface (adapted from^20^).

Two extracted human maxillary premolars were examined in the study. The teeth were extracted for orthodontic reasons and were used with the patients’ written informed consent and permission to utilize the obtained data for research purposes. The primary criteria for the teeth selection were as follows: (1) intact buccal enamel with no white spots, signs of dental fluorosis or enamel hypoplasia; (2) no pre-treatment with any chemical agents (such as hydrogen peroxide); (3) no previous orthodontic, endodontic or restorative treatment; (4) specimens correctly stored following extraction. The secondary criterion for the teeth selection was the presence of visible and invisible EMCs on the outer buccal enamel surface (Fig. 2)^6,20,42^. The protocol of the study was approved by the ethical review board of Vilnius University (Lithuania) and all the experiments were performed in accordance with relevant guidelines. The teeth were prepared in accordance with the guidelines of the International Organization for Standardization (ISO/TS 11405; 2003)^43^.

**Figure 3:**
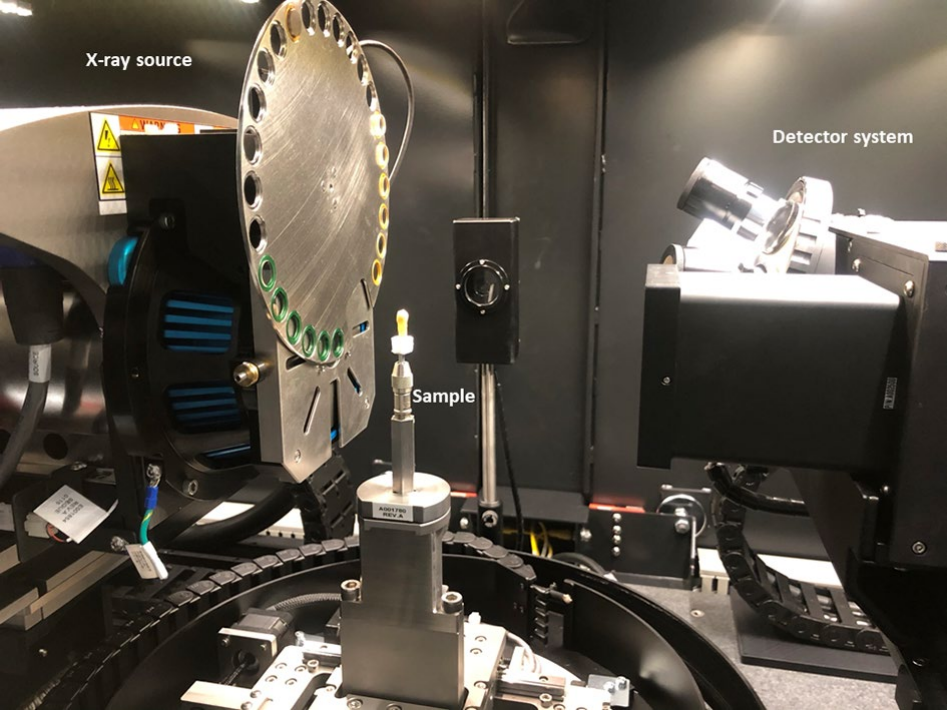
The general layout of the experiment including the X-ray source, the specimen, and the detector.

ZEISS Xradia 520 Versa X-ray microscope (Pleasanton, CA 94588, USA) was used in the study of a 3D distribution of cracks and other features in the teeth specimens. The general layout of the experiment including the X-ray source, the specimen, and the detector is shown in Fig. 3. The source-sample and sample-detector distances were adjusted to achieve the maximum magnification with the full field-of-view of the tooth specimen. For this experiment we used the following distances: source-sample = 21 mm and sample-detector = 125 mm. The sample projection images were acquired in absorption mode using geometrical magnification and employing a CCD detector resulting in a detector size of 2048 $\times$ 2048 pixels. To achieve the optimal signal-to-noise level (intensities of $>5000$ grey value over low transmission regions), the exposure time of 5 s was chosen. The source, sample and detector distances resulted in a $5 \times 5 \times 5\, \upmu \hbox {m}^{3}$ voxel size which in this case defines the experimental resolution. The tungsten source emits mostly continuous (bremsstrahlung) spectrum of the X-ray radiation. The transmission through the specimen was optimized by narrowing down the X-ray bandwidth using low energy filter (520 Versa LE4 filter) and the applied voltage. The projection scans were collected with the X-ray source settings of at 80 kV and 7 W. The optimal transmission of 30–35% was achieved and 1601 2D projection scans were collected over ${360}^{\circ }$ sample rotation.

The acquired tomography datasets were reconstructed using a Reconstructor software (ZEISS). The reconstructed 3D volume resulted in 2048 slices of 2048 $\times$ 2048 pixel 2D images. The 3D reconstructed datasets were analyzed and segmented using Dragonfly (Object Research Systems) software. To separate crack, dentin, and enamel volumes a Deep Learning (DL) algorithm, part of the Dragonfly’s segmentation toolkit, was successfully utilized. For our segmentation needs we implemented Dragonfly’s pre-built UNet neural network^44^. Due to dentin density variation simple thresholding did not result in a satisfactory crack separation. In this case the DL algorithm provided superior feature recognition as compared to other segmentation methods.

## Results

### Visualization of enamel microcrack

**Figure 4:**
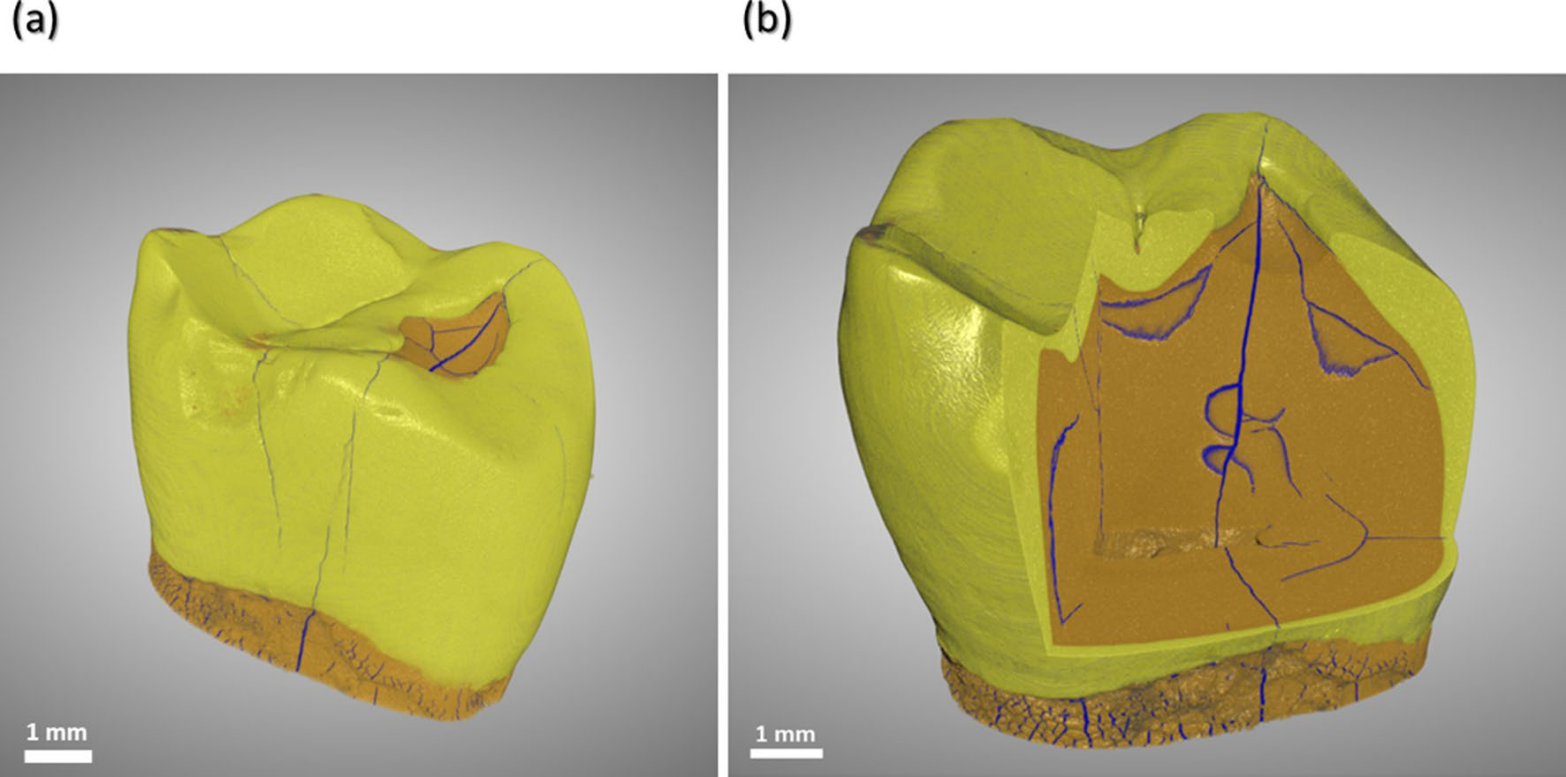
Scanned image (**a**) of the entire tooth using X-ray $\mu$CT and (**b**) a 3D virtual cut through a segmented tooth showing cracks (blue) inside the enamel (yellow) and the dentin (brown; see Supplementary Video S1).

**Figure 5:**
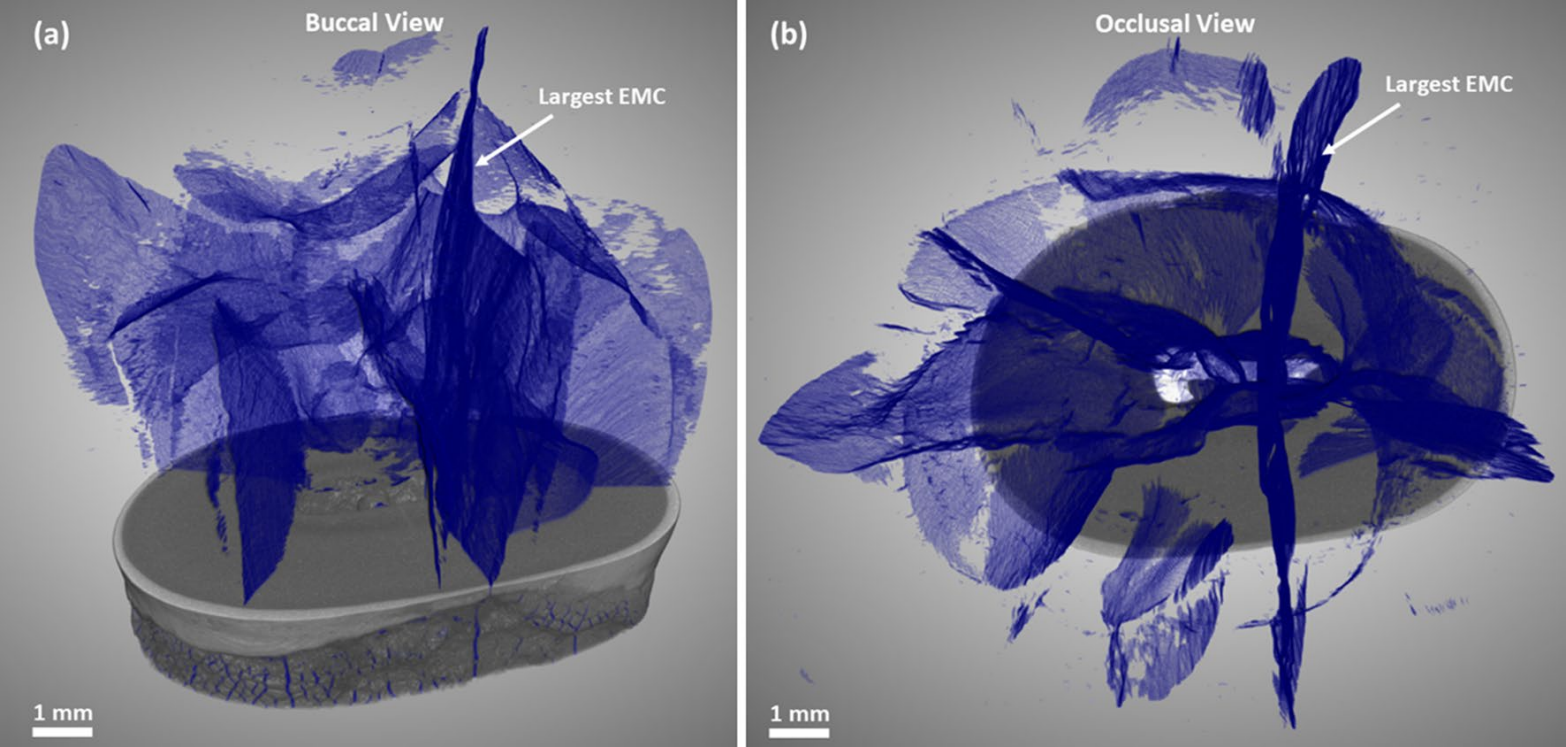
3D virtual cut through the tooth showing an intricate network of cracks (blue): (**a**) buccal view and (**b**) occlusal view. The largest EMC is marked.

X-ray $\mu$CT used in the study allowed to visualize and distinguish both the entire tooth and the smaller structures within it, such as cracks in three dimensions (Fig. 4). The scanning technique used made it possible to recognize and detect not only those EMCs that are visible on the outer surface but also those that are buried deep inside the tooth. The segmented network of cracks within the 3D volume of the scanned tooth is shown in Fig. 5.

### Evaluation of enamel microcrack dynamics

**Figure 6:**
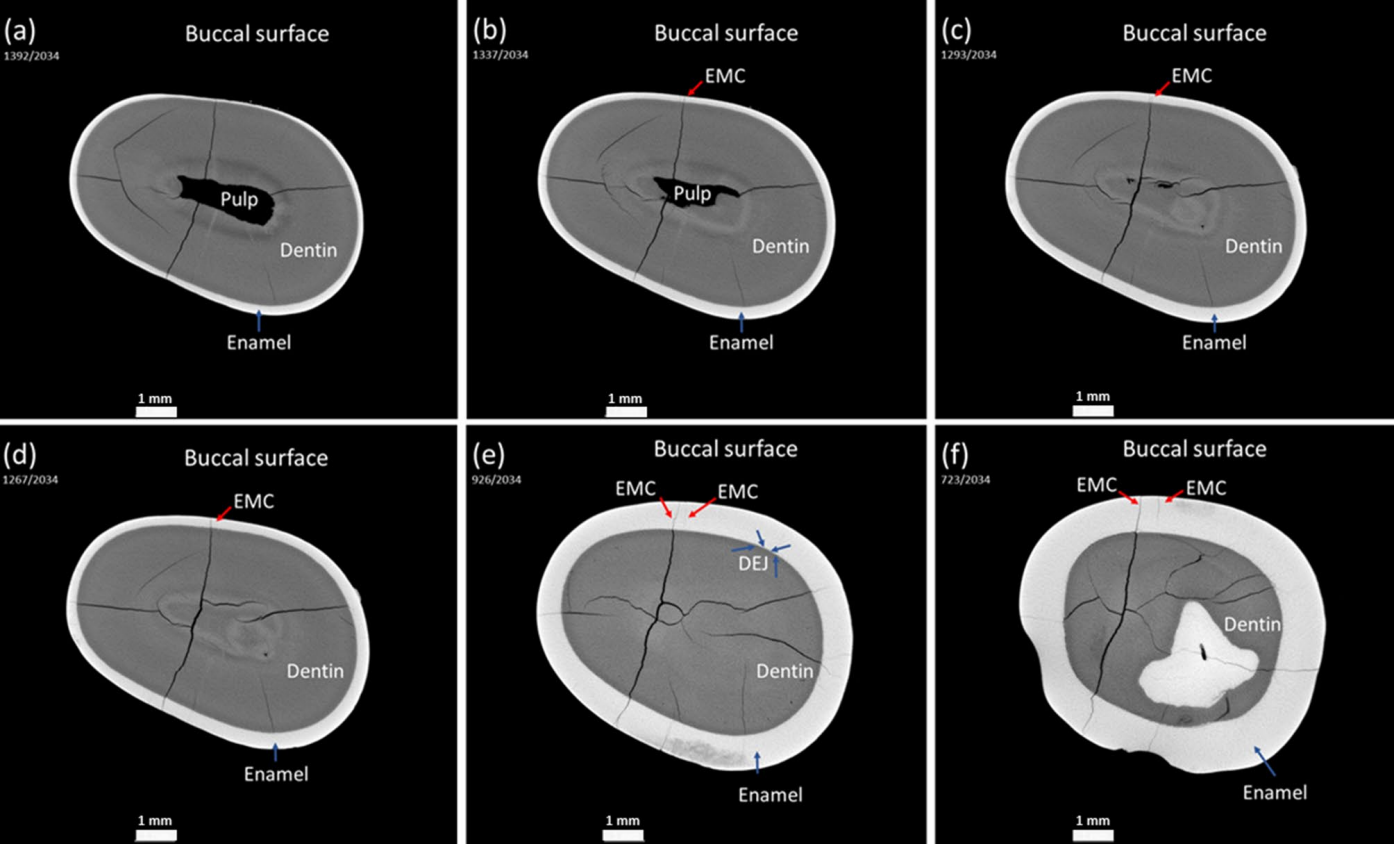
The path of the longest EMC invisible on the outer buccal surface as it extends from (**a**,**b**) the cervical (**c**,**d**) to the middle and (**e**,**f**) occlusal parts of the tooth crown. Anatomical tooth structures (pulp, dentin, DEJ, enamel, EMC) are marked.

**Figure 7:**
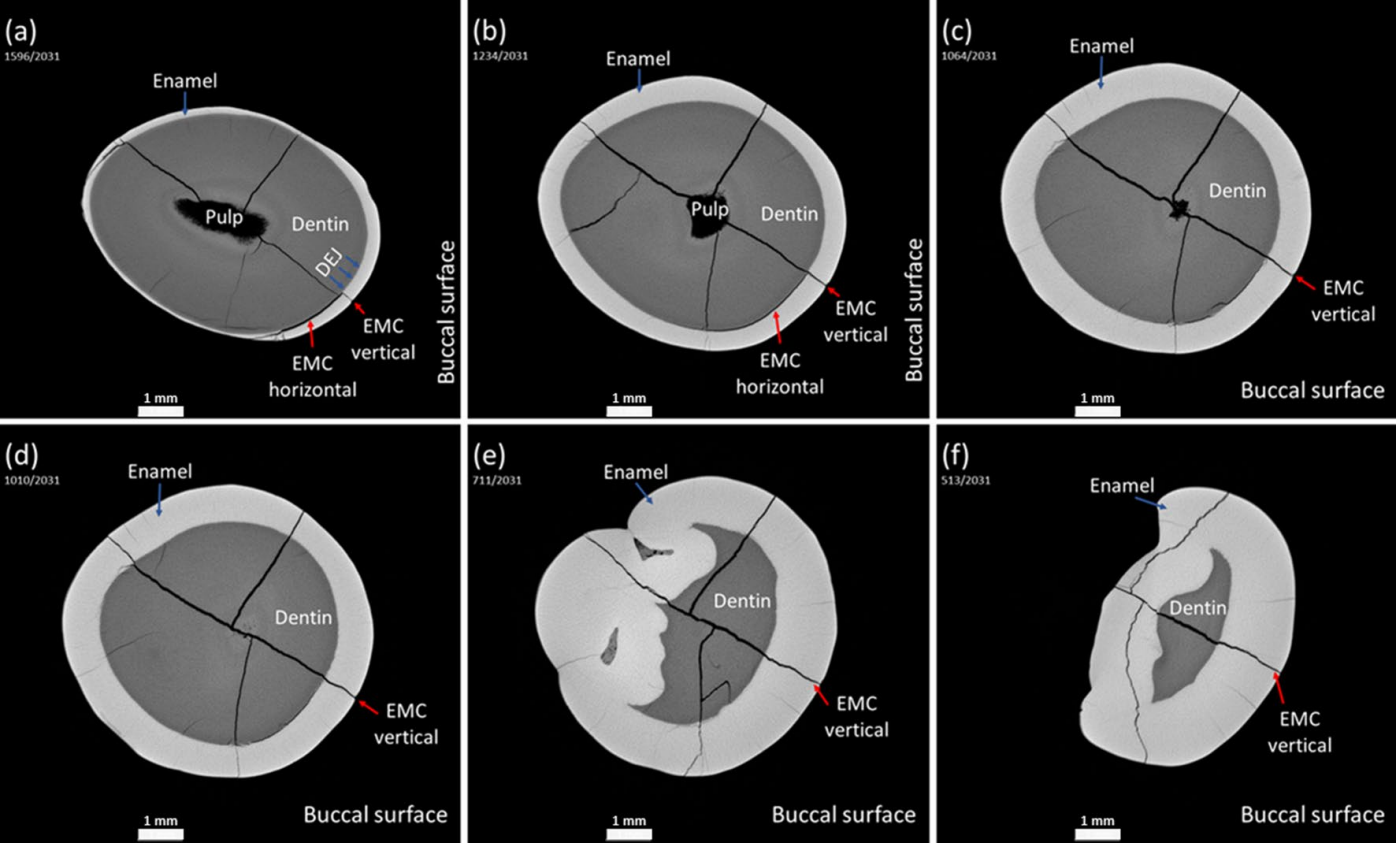
The path of the longest EMC visible on the outer buccal surface as it extends from (**a**,**b**) the cervical (**c**,**d**) to the middle and (**e**,**f**) occlusal parts of the tooth crown. Anatomical tooth structures (pulp, dentin, DEJ, enamel, EMC) are marked.

The 3D visualization, combined with DL assisted segmentation, allowed us to evaluate the dynamics of EMC. Path of the EMC from the outer surface towards the inner tooth structures, such as the dentin and the pulp, can be easily monitored in three-dimensions in the cervical, middle, or occlusal part of the premolar (Figs. 6, 7). The morphological characteristics and behavior of EMC could be accurately examined in a quantitative manner even in those areas where the layer of enamel is the thinnest. The convexity of the buccal premolar surface, especially in the middle part of the tooth, did not affect the procedure of the visualization and evaluation of EMCs. Teeth with EMCs visible on the outer surface and teeth without such EMCs were examined in the same way, but visible EMCs appeared to be more pronounced in either cervical, middle, or occlusal part of the tooth (Figs. 6, 7).

### Enamel microcrack position with respect to dentin-enamel junction

**Figure 8:**
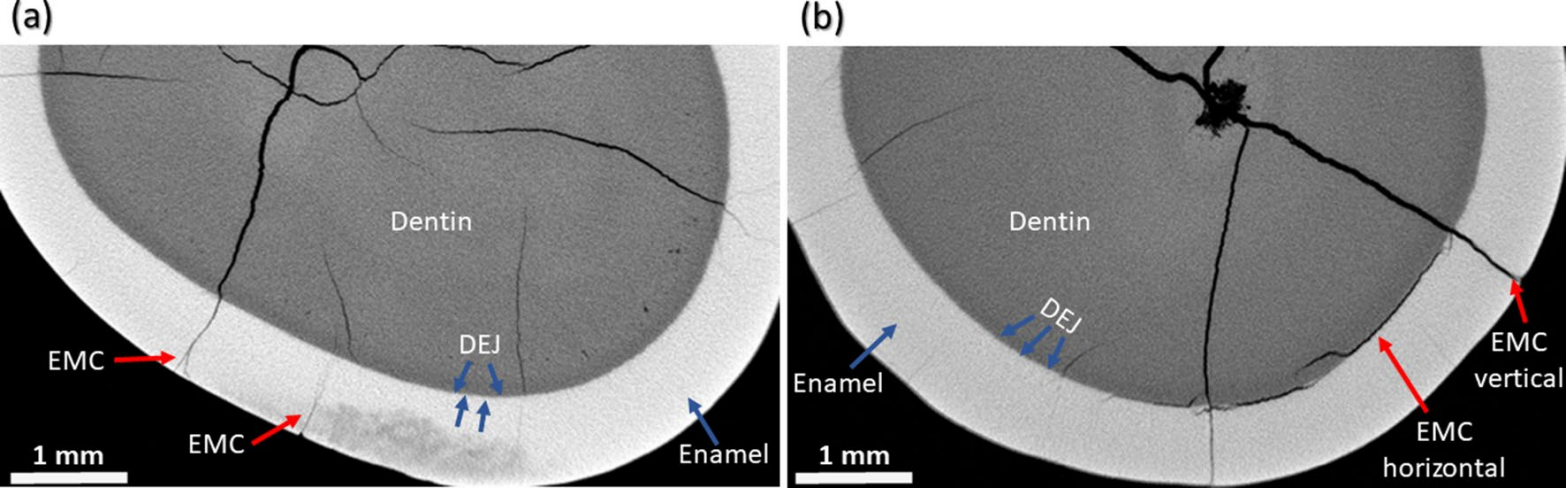
The position of an EMC with respect to the DEJ when the EMC is (**a**) invisible and (**b**) visible on the outer buccal surface. Anatomical tooth structures (dentin, DEJ, enamel, EMC) are marked.

The applied scanning technique allowed to accurately and non-destructively assess the position of an EMC with respect to the DEJ; it made it possible to determine whether the EMC stopped at the DEJ or crossed the boundary between the enamel and the dentin (Fig. 8).

## Discussion

The results of this study demonstrate that X-ray $\mu$CT can be considered as a valuable technique for the recognition and detection of EMCs (crack parameters measured by SEM, laser ultrasonic system, and ultrasound: length range, 0.24–10.15 mm; width range, 0.25–${35.04}{\, \upmu \hbox {m}}$; depth range, ${0.10\pm 0.01}\, \hbox {mm}$ of the craze lines, 0.80–1.00 mm in the cracked tooth, or $\approx$ 1.2 mm depth of the crack in simulated dentin)^20,22,23^. Compared to the 3D scanning methods used so far (OCT, ultrasound), this technique allows evaluation of the damage to the tooth structure in the area of EMC directly (i.e. not through replication procedure), precisely, and with micrometer resolution^21,22,31,32^. Moreover, this technique does not require sample preparation (i.e. selection of appropriate coupling media for teeth enamel) and vacuuming, and is not sensitive to non-flat surfaces ( apart from incisors, the remaining groups of teeth have a convex vestibular surface)^21,22,31,32^. One of the most important advantages of this method is the ability to look at the deeper layers of tooth tissue without destroying the sample. It is important to emphasize that the scanning procedure does not require additional sample handling and is performed only by rotating the specimen illuminated by the X-ray beam. This is especially relevant when human teeth are used for the examination. Due to great values of hardness ($\approx$ 3–6 GPa), elastic modulus ($\approx$ 70–120 GPa), lower fracture toughness (outer enamel (at the tooth’s surface) ${0.67\pm 0.12}\, \hbox {MPa m}^{0.5}$, inner enamel ${2.62\pm 1.39}\, \hbox {MPa m}^{0.5}/\hbox {mm}$), and high brittleness of the outer enamel, cutting of samples could weaken the tooth structure and result in greater EMCs than before the samples preparation procedure^45–47^.

The applied technique (X-ray $\mu$CT in combination with DL assisted segmentation) enabled the examination of EMC dynamics as the latter extends from the cervical to the occlusal part of the tooth. The results of the present study showed that the pattern of EMC propagation was different depending on its location on the tooth surface. In the cervical region, where enamel is the thinnest ($\approx$ 0.5 mm), the contour of the crack extending into deeper tooth layers was barely visible or a clear arrest of the EMC at the DEJ was noticed^45^. Approaching the occlusal tooth region with the largest enamel thickness (up to $\approx$ 2.5 mm near the cusp for the molar teeth) and the greatest distance to the pulp, the boundary between the enamel crack and the dentin crack became invisible^45^. It is interesting to note that in the cervical region of the tooth with EMC visible on the outer surface a pronounced horizontal crack bordering on the vertical EMC and extending along the DEJ was observed (Fig. 7a). Studies have shown that the internal enamel (the enamel near the DEJ where the rods extend within groups that are obliquely oriented to one another) demonstrates strong resistance to fracture and that crack growth resistance increases from outside to inside^47–50^. The rise in crack growth resistance within the inner enamel can be explained by several mechanisms of toughening, including crack bridging, crack deflection, and microcracking (i.e. the ability of the enamel microstructure to promote guided crack growth and arrest)^47^. Thus, it can be hypothesized that the apparent crack branching (or additional cracks) and a clear arrest of an EMC at the DEJ in the cervical region, where the tooth is more prone to damage due to the thinner enamel layer and shorter distance to the dentin and the pulp, are signs of tooth self-defense.

One of the objectives of the research was to demonstrate that the proposed technique can be used to recognize and detect EMCs of different severity. Employing X-ray $\mu$CT, combined with DL assisted segmentation, allowed for the visualization of EMCs, both those which are visible on the outer surface and also those that are buried deep inside the tooth. Visible EMCs appeared to be more pronounced in all parts of the tooth. This finding is supported by a previously published study demonstrating that in the majority of the examined cases teeth with pronounced EMCs possessed higher mean overall length and width values compared to those with invisible EMCs^20,51^.

In this work we have validated an X-ray $\mu$CT as a method suitable for the 3D qualitative analysis of EMCs with distinct precision and versatility. The proposed approach is fully expandable of the teeth microstructure analysis in a quantitative manner. This opens up new possibilities to measure the EMC depth parameter, as no such study has been carried out directly before.

## Conclusions

The proposed technique—using X-ray $\mu$CT in combination with DL assisted segmentation—offers a powerful method for 3D non-destructive visualization of EMCs and could be applied on all teeth with cracks of different severity. This technique allows 3D monitoring of EMC dynamics at various tooth points regardless of surface convexity and relief, enamel thickness at a particular examination site, and distance to the DEJ. For the first time to our knowledge, the extent of tooth damage in the area of EMC was examined using an experimentally validated 3D non-destructive research method, this way opening new opportunities in the field of interdisciplinary research and indicating a prospective future in vitro and in vivo application of the technique.

The wider possibilities of EMCs’ examination provide an opportunity to supplement the 2D information about cracks available in the literature so far and presuppose a change or extension of the existing definition of EMCs, to broaden the understanding of the potential risks of these cracks, and to develop algorithms for the treatment of teeth with EMCs in clinical practice.

## Supplementary Information

Supplementary Information 1.

Supplementary Legend.
