# Supplementary material for: Three-dimensional non-destructive visualization of teeth enamel microcracks using X-ray micro-computed tomography
Source: Sci Rep. 2021 Jul 20;11:14810. doi: 10.1038/s41598-021-94303-4 (PMC8292334; doi:10.1038/s41598-021-94303-4)
Supplement: Supplementary file 2 — Supplementary Legend. [file 41598_2021_94303_MOESM2_ESM.pdf]

Legend for the supplementary file

Scanning of the entire tooth using ZEISS Xradia 520 Versa X-ray microscope (Pleasanton, CA 94588, USA).
